# Bidirectional Effects Between Parental Care and Depression Among Adolescent Boys: Results From the Chinese Family Panel Studies

**DOI:** 10.3389/fpsyg.2022.803450

**Published:** 2022-06-15

**Authors:** Jingyu Wang, Jian Jiao

**Affiliations:** ^1^School of Languages and Communication Studies, Beijing Jiao Tong University, Beijing, China; ^2^Department of Communication, University of Arizona, Tucson, AZ, United States

**Keywords:** adolescence, parenting, parental care, depression, gender

## Abstract

**Background:**

Research has consistently shown the adverse effects of inappropriate parenting on adolescent depression. Meanwhile, interpersonal theories of depression suggest that depressed individuals elicit frustration and rejection from their relational partners.

**Method:**

Using two-wave data from the Chinese Family Panel Studies (CFPS), the present study examined the prospective relationships between parental care and adolescent depression. Participant were 426 adolescents (54.9% male) born in 1999 (ages at T1 and T2 were 11 and 13).

**Results:**

Results from the structural equation model showed that parental care prospectively and negatively predicted depression among both adolescent boys and girls. Inversely, adolescent boys’ depression, but not girls’ depression, negatively predicted subsequent parental care.

**Conclusion:**

The results suggest the interactive dynamic between parental care and adolescent depression as well as parents’ gendered responses to adolescent depression.

## Introduction

Depressive disorders include a wide range of categories (e.g., disruptive mood dysregulation and major depressive disorder), and a shared feature of all depressive disorders is “the presence of sad, empty, or irritable mood, accompanied by somatic and cognitive changes that significantly affect the individual’s capacity to function” (American Psychiatric Association, 2013). Adolescence is a developmental period that features a heightened risk for depression, caused by a multitude of biological, psychological, and contextual factors surrounding this transition period (Thapar et al., 2012; Rudolph and Flynn, 2014; Lu, 2019). Moreover, the onset of depression during adolescence tends to perpetuate into later life stages, leading to enduring difficulties in one’s personal and social lives (Lewinsohn et al., 2003; Clayborne et al., 2019).

A variety of factors have been found to trigger depressive symptoms in adolescents, among which is poor family functioning (Sander and McCarty, 2005; Alloy et al., 2006; Freed et al., 2016). Indeed, although peer relationships become more salient during adolescence, parents continue to play a crucial role in adolescent development (Steinberg and Morris, 2001). In particular, a few family factors have been found to increase the risk for adolescent depression, such as aversive family environments (e.g., high conflict and low cohesiveness; Sheeber et al., 2001), inappropriate parenting (e.g., parental rejection and lack of authoritative parenting; McLeod et al., 2007; Lu, 2019), and low-quality parent–child relationships (e.g., insecure attachment representations; Allen et al., 2007; Spruit et al., 2020). The present study aimed to understand the prospective relationships between parental care and adolescent depression and explore gender differences in these relationships relying on data collected from a national and representative sample of early Chinese adolescents.

### Lack of Parental Care as an Antecedent to Child Depression

Abaied and Rudolph (2014) argue that the effects of family dysfunction on adolescent depression is achieved through disrupting the appropriate socialization processes of emotion regulation. Indeed, the effects of emotionally negative parenting (e.g., low parental care and high parental psychological control) are more consequential than the effects from parental physical or sexual abuse in predicting adolescent depression (e.g., Segrin, 2011). With regard to parental care, research has consistently revealed low parental care as a risk factor for child depression and a lifetime history of depression along with other mental health disorders (e.g., Narita et al., 2000; Enns et al., 2002; Yap et al., 2014). In the Chinese context, the concurrent and inverse association between parental care and child depression is also well documented (e.g., Gao et al., 2012; Zhang et al., 2019). Additionally, in a longitudinal investigation relying on a regional sample, Fan et al. (2018) found the prospective effect of parental care on child depression. Thus, the first purpose of the present study was to examine the prospective effect of parental care on adolescent depression using a larger and more representative sample. The following hypothesis was advanced:

*H1:* Parental care prospectively and negatively predicts adolescent depression.

### Lack of Parental Care as a Possible Consequence of Child Depression

The argument that child traits elicit particular parenting practices has been broadly examined in a variety of domains (e.g., Brinke et al., 2017; Pinquart, 2017). Indeed, Parker et al. (1979) argued that parent–child bonds are influenced by both child and parent traits as well as the reciprocal and evolving relationship between them. Similarly, Belsky (1984) argued that parenting is impacted by multiple factors, including parent personality and psychological wellbeing, child characteristics, and contextual stress and support sources.

With regard to depression, a few theoretical perspectives have argued that depressed individuals tend to create a stressful interpersonal environment which in turn may contribute to the maintenance and exacerbation of their current depressive symptoms and increase their vulnerability to future depression (e.g., Coyne, 1976a; Liu and Alloy, 2010; Hames et al., 2013). As opposed to assuming the passive role of depressed individuals in their environment, these theories emphasize individuals with depression as an active member in shaping their environment. Interpersonal theory of Coyne (1976a,b) of depression argues that depressed individuals excessively seek reassurance and induce negative affect in their relationships, which in turn elicit frustration, avoidance, and rejection from others (e.g., Segrin and Dillard, 1992; Joiner, 1994; Stewart and Harkness, 2017). Stress-generation theory of Hammen (2006) articulates that depressed individuals tend to generate interpersonal stress and conflict, which in turn may elicit interpersonal rejection and subsequent depression. With particular attention to the family context, having a depressed child may be an emotional burden for parents, which may in turn impair parent–child relationships (e.g., Meltzer et al., 2011; Wilkinson et al., 2021). Given these negative interpersonal effects of depression, the second purpose of the present study was to examine the prospective effect of adolescent depression on parental care. Indeed, longitudinal studies have produced mixed findings concerning the prospective relationships between child depression and some other parenting styles and dimensions (e.g., Sheeber et al., 1997; Nolan et al., 2003; Needham, 2008). For example, Needham (2008) found a bidirectional relationship between parental support and adolescent depression. Nolan et al. (2003) found the ill effect of parental rejection on adolescent depression but not vice versa. In a recent meta-analytic study investigating the associations between parenting and child internalizing problems (e.g., anxiety and depression), Pinquart (2017) reported bidirectional effects between child internalizing problems and parental warmth, psychological control, and authoritative parenting. In light of the interpersonal theories of depression, the following hypothesis was advanced:

*H2:* Adolescent depression prospectively and negatively predicts parental care.

### Gender Differences in the Dynamics Between Depression and Interpersonal Relations

Previous research has also examined gender differences in the associations between depression and its surrounding interpersonal dynamics (e.g., Sheeber et al., 1997; Conway et al., 2011). For example, in peer contexts, only among adolescent girls, depression prospectively leads to low friendship quality and stability (Prinstein et al., 2005; Rudolph and Flynn, 2007). Similarly, three-wave longitudinal study of Rudolph et al. (2009) found that depression triggers interpersonal stressors which in turn perpetuates depression among adolescent girls but not boys. In the family context, Sheeber et al. (1997) observed the negative effects of less supportive and more conflictual family environments on adolescent depression but not vice versa among both boys and girls. Similarly, Schwartz et al. (2012) found the prospective effect of positive parental behaviors on depression among both adolescent boys and girls. Thus, it appears that adolescent girls suffer more relational consequences engendered by their depression in peer contexts, but the dynamics appear comparable between adolescent boys and girls in the family context. Consequently, the third purpose of the present study was to examine gender differences in the prospective relationships between parental care and adolescent depression. The following research question was proposed:

*RQ:* Are there gender differences in the prospective relationships between parental care and adolescent depression?

**Figure 1:**
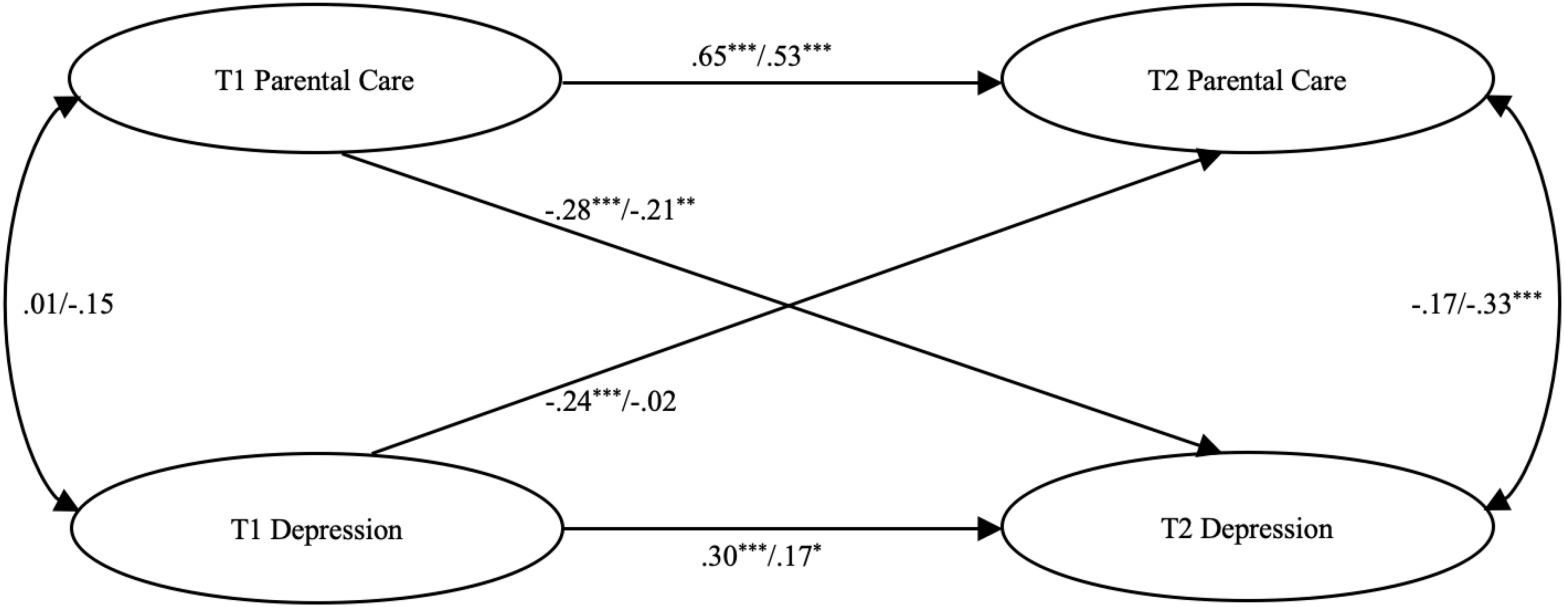
Longitudinal structural equation model (SEM) model between parental care and adolescent depression. T1: CFPS data from 2010; T2: CFPS data from 2012. The values are correlations and standardized regression coefficients. Values to the left of the slash are for male participants and to the right of the slash are for female participants. Factor loadings for each latent construct can be found in Table 4. ^*^p < 0.05, ^**^p < 0.01, ^***^p < 0.001.

To summarize, Figure 1 represents a theoretical model between parental care and adolescent depression. In particular, building upon previous research that reveals gendered and bidirectional dynamics between depression and interpersonal relations, it appears plausible that parental care has an inverse effect on adolescent depression and vice versa that adolescent depression also has an inverse effect on parental care. Thus, relying on a nationally representative sample, the present study aimed to explicate the prospective associations between parental care and adolescent depression and explore if any gender differences exist in these associations in the Chinese context.

## Materials and Methods

### Participants and Procedure

Data for this study were retrieved from the Chinese Family Panel Studies (CFPS) which is a large national survey that collects data every other year since 2010 (still ongoing; Xie and Hu, 2014). CFPS collects data through personal visits (data recorded *via* computer-assisted personal interviewing technology) on the individual (children and adults separately), family, and community levels across 25 provinces in China that cover 95% of the Chinese population (not including Hong Kong, Macau, and Taiwan). Its sampling procedure followed systematic sampling with multiple stages and implicit stratification (Xie and Lu, 2015). In particular, the sampling process involved three stages; that is, sampling districts (if urban) and counties (if rural) from the 25 provinces, sampling communities from selected districts and counties, and sampling households from selected communities. Moreover, to increase sample representativeness to the entire Chinese population, the research team used urban/rural divide and socioeconomic scores to stratify the sampling units. For more details about the sampling techniques used, please see Xie and Lu (2015).

Data used in the present study were from the CFPS 2010 (T1) and 2012 (T2) child datasets. These two datasets were selected because in 2010 and 2012, participants answered the parental care and depression questions at each of the two waves of collection, enabling longitudinal analyses. Since 2012, all participants only responded to the parental care and depression questions only once over the years of their participation. Thus, only the 2010 and 2012 datasets could be used for longitudinal analyses on parental care and depression. The 2010 and 2012 datasets include 8,990 and 8,620 cases aged below 18. Because only participants born in 1999 answered the questions about parental care and depression at both T1 and T2, the present study only included these children, resulting in sample sizes of 567 and 548 for T1 and T2 separately. Nine cases from T1 and 53 cases from T2 were removed as they completely missed the questions on parental care and depression. After this removal, these two datasets were matched and combined by participant ID, resulting in a final sample of 426 participants from 25 provinces (see Table 1 for the data filtering process). The participants (54.9% males) were 11 at T1 and 13 at T2.

**Table 1:** Data filtering process.

	T1	T2
Step 1: Entire dataset	*n* = 8,990	*n* = 8,620
Step 2: Born in 1999	*n* = 567	*n* = 548
Step 3: Provided data on study variables	*n* = 558	*n* = 495
Step 4: Provided data on study variables at both T1 & T2	*n* = 426	*n* = 426

T1: CFPS data from 2010; T2: CFPS data from 2012.

### Measures

Participants completed the following measures in Chinese.

#### Parental Care

The Parental Bonding Instrument (PBI; Yu et al., 2014) was used to assess parental care at both T1 and T2. This instrument was created by referencing Parental Bonding Instrument of Parker et al. (1979). Differing from scale of Parker et al. (1979) that measures two aspects of parenting (i.e., parental care and overprotection), the instrument used in this study only assesses parental care that features parental attention to and involvement into their children’s lives and frequent parent–child interactions. Participants indicated how often their parents engaged in certain parenting behaviors on a 5-point Likert scale (1 = *never*, 5 = *always*). Sample items include “My parents spoke to me with a warm and friendly voice” and “My parents enjoyed talking things over with me.” One item (i.e., “My parents criticized me”) from the original 14-item scale was dropped due to its non-significant factor loading on the latent construct of parental care observed in the present study and by the CFPS research team (Yu et al., 2014). Cronbach’s *α* of the final 13-item scale was 0.85 at both T1 and T2. A higher score on each item indicates a higher level of parental care.

#### Depression

At T1, the Kessler 6 Distress scale (Kessler et al., 2003) was used to assess adolescent depression. Participants indicated how they had been feeling in the past 30 days on a 5-point Likert scale (1 = *almost every day*, 5 = *never*). Sample items include “During the past 30 days, about how often did you feel so depressed that nothing could cheer you up?” and “During the past 30 days, about how often did you feel worthless?.” Cronbach’s *α* of the 6-item scale was 0.79. Scores for all the items were reversed so that a higher score indicates greater depression. At T2, the Center for Epidemiologic Studies Depression scale (Radloff, 1977) was used to assess adolescent depression. Participants indicated how they felt in the past week on a 4-point Likert scale (1 = *almost never*, 4 = *most of the days*). Sample items include “I felt sad”++ and “I felt fearful.” Cronbach’s *α* of the 20-item scale was 0.85. After reversing scores for four items, a higher score on each item indicates greater depression.

## Results

The free statistical software R 4.0.3 (R Core Team, 2020) and the Lavaan package (Rosseel, 2012) were used to evaluate the confirmatory factor analysis (CFA) models and the subsequent structural equation model (SEM). There were 0.6% missing values in the final dataset, and full information maximum likelihood was used for imputation (Enders and Bandalos, 2001). Maximum likelihood was used to estimate the parameters (Rossi, 2018).

### Descriptive Analysis

One of the purposes of this study was to investigate gender differences in the associations between parental care and adolescent depression. Table 2 shows the means, standard deviations, and independent *t*-test results on parental care and adolescent depression (treated as manifest variables; each of the four manifest variables was created by averaging the scores from its corresponding scale) for male and female participants at T1 and T2. The results showed no gender differences in the levels of parental care and adolescent depression at either T1 or T2. Table 3 shows the bivariate correlations between the study variables (treated as manifest variables) for male and female participants separately. The associations observed here were further examined in the final SEM model.

**Table 2:** Descriptive analysis between male and female participants.

Variable	Range	Male participants (*n* = 234)		Female participants (*n* = 192)		*t*	*p*
		*M*	SD	*M*	SD		
1. T1 Parental care	1.15–4.85	3.06	0.69	3.11	0.63	−0.81	*ns*
2. T2 Parental care	1.46–4.92	3.12	0.64	3.20	0.58	−1.37	*ns*
3. T1 Depression	1–4	1.41	0.59	1.48	0.66	−1.15	*ns*
4. T2 Depression	1–2.74	1.51	0.35	1.48	0.33	1.00	*ns*

T1: CFPS data from 2010; T2: CFPS data from 2012.

**Table 3:** Bivariate correlations between study variables.

Variable	1	2	3	4
1. T1 Parental care	–	0.47^***^	−0.14	−0.21^**^
2. T2 Parental care	0.54^***^	–	−0.07	−0.33^***^
3. T1 Depression	0.02	−0.18^**^	–	0.19^**^
4. T2 Depression	−0.24^***^	−0.29^***^	0.24^***^	–

T1: CFPS data from 2010; T2: CFPS data from 2012. Values below the diagonal are for male participants, and above the diagonal are for female participants.

** *p* < 0.01;

*** *p* < 0.001.

### Confirmatory Factor Analysis

Prior to assessing the prospective relationships between parental care and adolescent depression, a measurement model with four latent constructs (i.e., parental care and adolescent depression at T1 and T2) was evaluated with CFA for its fit to the sample date. To reduce the potential for error due to the estimation of over-identified latent constructs, three parcels were created using the balancing technique when representing the latent constructs as advocated by Little et al. (2002). In this CFA model, the scales for the four latent constructs were set by constraining their latent variances to 1.0 and means to 0.0 (Brown, 2006). All factor loadings were freely estimated (see Table 4). This CFA model demonstrated an adequate model fit to the data, *χ*^2^(48) = 108.887, *p* < 0.001, CFI = 0.973, RMSEA = 0.055, 90% CI [0.041, 0.068], SRMR = 0.035 (Little, 2013).

**Table 4:** Unstandardized and standardized factor loadings.

Latent construct	Unstandardized (SE)	Standardized
*T1 Parental care*		
Parcel 1	0.563 (0.033)	0.758
Parcel 2	0.610 (0.032)	0.813
Parcel 3	0.679 (0.032)	0.868
*T2 Parental care*		
Parcel 1	0.471 (0.027)	0.775
Parcel 2	0.623 (0.035)	0.794
Parcel 3	0.612 (0.035)	0.777
*T1 Depression*		
Parcel 1	0.578 (0.034)	0.793
Parcel 2	0.610 (0.038)	0.755
Parcel 3	0.486 (0.031)	0.742
*T2 Depression*		
Parcel 1	0.318 (0.017)	0.808
Parcel 2	0.310 (0.016)	0.825
Parcel 3	0.313 (0.017)	0.799

T1: CFPS data from 2010; T2: CFPS data from 2012. All factor loadings are significant at *p* < 0.001.

Prior to testing the gender differences in the prospective relationships between parental care and adolescent depression, a *χ*^2^ difference test was used to compare the unconstructed measurement model and the constrained model in which all the factor loadings and intercepts were set to be equivalent for male and female participants. Although the constrained model also showed an adequate model fit to the data, *χ*^2^(112) = 169.259, *p* < 0.001, CFI = 0.975, RMSEA = 0.049, 90% CI [0.033, 0.064], SRMR = 0.044. The results from the *χ*^2^ difference test showed no statistical difference between the unconstrained and constrained models, *χ*^2^ difference (64) = 60.371, *p* = 0.606. Thus, measurement equivalence across genders was confirmed prior to testing the gender differences in the associations between parental care and depression (Little, 2013).

To test the prospective relationships between parental care and adolescent depression by gender, an SEM model specifying the associations between all the four latent constructs (i.e., parental care and adolescent depression at T1 and T2) was tested. The model demonstrated an adequate model fit to the data, *χ*^2^(96) = 138.875, *p* < 0.01, CFI = 0.981, RMSEA = 0.046, 90% CI [0.027, 0.062], SRMR = 0.039 (see Figure 1). The results showed that parental care at T1 significantly and negatively predicted adolescent depression at T2 among both male, *b* = −0.31, SE = 0.08, *p* < 0.001, and female participants, *b* = −0.22, SE = 0.09, *p* = 0.01, controlling for depression at T1 (i.e., including T1 depression as a covariate in predicting T2 depression). In contrast, male participants’ depression at T1 significantly and negatively predicted parental care at T2, *b* = −0.34, SE = 0.10, *p* = 0.001, whereas female participants’ depression at T1 did not significantly predict parental care at T2, *b* = −0.02, SE = 0.09, *ns*, controlling for parental care at T1 (i.e., including T1 parental care as a covariate in predicting T2 parental care). Thus, H1 was supported, H2 was partially supported, and RQ was addressed that gender differences existed in the prospective relationships between parental care and adolescent depression.

## Discussion

The present study aimed to examine the prospective relationships between parental care and child depression at early adolescence in the Chinese context. The findings show that low parental care prospectively predicted adolescent depression 2 years later among both boys and girls, controlling for their initial levels of depression. Inversely, adolescent depression prospectively predicted low parental care among boys but not girls, controlling for initial levels of parental care.

Parental care prospectively and negatively predicted adolescent depression among both boys and girls. This finding is consistent with the literature that shows the aversive consequences of unhealthy family dynamics and parent–child bonding on adolescent depression (e.g., Abaied and Rudolph, 2014) and replicated the finding from Fan et al. (2018) revealing the ill effects of low parental care on child depression in the Chinese context. As a form of emotionally negative parenting, withholding parental care might make the child feel devalued and not worthy of love, which may in turn engender their depressive symptoms (e.g., Segrin, 2011). Indeed, longitudinal evidence shows self-esteem as an antecedent to depression (Orth et al., 2008). Future studies may examine if reduced self-esteem explains the association between low parental care and subsequent adolescent depression. Meanwhile, high levels of parental care were associated with low levels of subsequent depression such that parental involvement into children’s lives and frequent parent–child communication helped buffer the risks for adolescent depression.

Depression negatively predicted subsequent parental care among adolescent boys. This finding is consistent with the interpersonal theories of depression that depressed individuals tend to elicit avoidance and rejection from others in their environment (e.g., Coyne, 1976a; Hammen, 2006). It is also possible that the interpersonal consequences of depression (e.g., generating interpersonal stressors) might be more pronounced in family relationships due to the unavoidable parent–child interaction in the family. Indeed, previous research has found that child depression was contagious such that parents subsequently developed depressive symptoms (e.g., Raposa et al., 2011). Moreover, such negative effects of child depression might also exacerbate the severity of depression among children themselves, generating a vicious circle of child depression and family dysfunction (e.g., Nolan et al., 2003). Nonetheless, the interactive process of parental care and adolescent depression suggests that psychopathology programs can intervene through either directly treating child depression or indirectly encouraging more parental care to help depressed adolescents.

Although no gender differences emerged in the levels of parental care or adolescent depression, only boys’ depression, but not girls’ depression, predicted subsequent low parental care. This finding is consistent with the literature on parental emotion socialization (e.g., Root and Denham, 2010). In the family context, parents differentiate their expectations for and reactions to their children’s emotion expression and regulation as a function of child gender, and consequently children’s understanding of the appropriateness of emotion expression and regulation is socialized in the family (e.g., Root and Denham, 2010). Depression is primarily a form of emotional disorder, and sadness is one major feature of depression (e.g., Abaied and Rudolph, 2014). Regarding sadness regulation, parents tend to encourage the expression of sadness with their adolescent daughters but not sons (Cassano et al., 2007). Indeed, in Western cultures, showing sadness is deemed non-masculine among boys, and compared to girls, boys showing sadness is viewed more negatively (Siegel and Alloy, 1990). The finding from the present study may reveal that, in non-Western cultures as well, compared to adolescent girls, boys showing sadness was viewed more negatively by their parents evidenced by the consequence of boys, but not girls, receiving less parental care engendered by their depressive symptoms. This finding is concerning in that adolescent boys may be socialized to suppress their negative emotions (e.g., sadness) which leads to negative consequences in personal and social functioning (e.g., Srivastava et al., 2009). Overall, although depressed girls might suffer more than boys from interpersonal disturbances engendered by their depression in peer relationships (e.g., Prinstein et al., 2005), based on the results from this study, it appears that boys suffer more than girls from the consequences of their depression in the family context.

Several limitations of the present study and future directions should be noted. First, although this study had a large national sample, participants’ age was constrained to the same year. Future research should examine the observed relationships with a more diverse sample in terms of participants’ age to better understand the dynamics between parental care and depression during adolescence. Second, this study had data from two waves with a two-year interval. Future research should design different intervals with multiple waves of data collection to further examine the developmental changes in the dynamics between parental care and child depression. Third, this study assessed depressive symptoms in a general community sample. Given the heterogeneity of depressive disorders, future research should also examine how the observed relationships differ by distinct categories of depression. Fourth, T1 and T2 collections used two different depression scales, thus the prospective effect of parental care on adolescent depression might be inflated due to the enlarged measurement error and reduced consistency in depression over time. Future research should use consistent instruments to avoid this problem. Fifth, this study used reports from adolescents on their general experiences with parental care. Future research should use other methods (e.g., observations) and gather data from each parent to examine the observed relationships. Finally, since the data were collected over 10 years ago, the extent that participants’ responses reflected today’s family dynamics and correspondingly the applicability of the findings are indeterminable.

Notwithstanding these limitations, the present study extended previous literature by showing the bidirectional and gendered effects between parental care and adolescent depression in the Chinese context. In particular, although son preference remains a unique feature of the Chinese culture (e.g., Eklund, 2015; Wang et al., 2020), results from the current study showed that parents were more tolerable with their daughters’ depressive symptoms as evidenced by the association between adolescent boys’, but not girls’, depressive symptoms and subsequent low parental care they received. Moreover, considering the reciprocal effects between parental care and depression among adolescent boys, policymakers and social workers should pay close attention to the family environment where the child struggles with mental health issues and encourage more positive parenting practices to help the child and family cope with these issues.

## Data Availability Statement

The datasets presented in this study can be found in online repositories. The names of the repository/repositories and accession number(s) can be found at: https://opendata.pku.edu.cn/dataverse/CFPS?language=en.

## Ethics Statement

The studies involving human participants were reviewed and approved by Peking University Biomedical Ethics Committee. Written informed consent to participate in this study was provided by the participants’ legal guardian/next of kin.

## Author Contributions

JW contributed to study conception and manuscript write-up. JJ contributed to study conception, data analysis, and manuscript write-up. All authors contributed to the article and approved the submitted version.

## Funding

This research was supported by the Fundamental Research Funds for the Central Universities 2022JBWG011.

## Conflict of Interest

The authors declare that the research was conducted in the absence of any commercial or financial relationships that could be construed as a potential conflict of interest.

## Publisher’s Note

All claims expressed in this article are solely those of the authors and do not necessarily represent those of their affiliated organizations, or those of the publisher, the editors and the reviewers. Any product that may be evaluated in this article, or claim that may be made by its manufacturer, is not guaranteed or endorsed by the publisher.
